# Two new species of frogs of the genus *Pristimantis* from Llanganates National Park in Ecuador with comments on the regional diversity of Ecuadorian *Pristimantis* (Anura, Craugastoridae)

**DOI:** 10.3897/zookeys.593.8063

**Published:** 2016-05-26

**Authors:** María J. Navarrete, Pablo J. Venegas, Santiago R. Ron

**Affiliations:** 1 Museo de Zoología, Escuela de Biología, Pontificia Universidad Católica del Ecuador, Av. 12 de Octubre y Roca, Aptdo. 17-01-2184, Quito, Ecuador; 2 División de Herpetología-Centro de Ornitología y Biodiversidad (CORBIDI), Santa Rita N˚105 Of. 202, Urb. Huertos de San Antonio, Surco, Lima, Perú

**Keywords:** Andes, Pristimantis llanganati sp. n., Pristimantis yanezi sp. n., species richness, systematics, taxonomy, Terrarana

## Abstract

We describe two new species of frogs of the genus *Pristimantis* from the eastern slopes of the Ecuadorian Andes, at Parque Nacional Llanganates. The new species are characterized by the spiny appearance typical of several species inhabiting montane forests. *Pristimantis yanezi* **sp. n**. is most similar to *Pristimantis colonensis* and *Pristimantis incanus* but differs from both in groin coloration and by having smaller tubercles on the upper eyelids, heels, and tarsus. *Pristimantis llanganati* **sp. n**. is most similar to *Pristimantis eriphus* and *Pristimantis chloronotus*. It can be distinguished from *Pristimantis eriphus* by the color pattern on the scapular region and by having smaller conical tubercles on the dorsum. *Pristimantis chloronotus* differs from *Pristimantis llanganati* **sp. n**. in having a pair of sinuous paravertebral folds. Both new species occur in a region with few amphibian collections and nothing is known about their abundance and ecology. Therefore, it is recommended to assign them to the Data Deficient Red List category. Updated figures of species richness of *Pristimantis* among biogeographic regions in Ecuador are also presented. *Pristimantis* reach their highest diversity in Montane Forests of the eastern versant of the Andes. Its species richness across regions cannot be explained by regional area, elevation, temperature, or precipitation. Political endemism in *Pristimantis* is higher than that of other terrestrial vertebrates.

## Introduction

With 484 species, *Pristimantis* is the most diverse genus of amphibians (Lynch and Duellman 1997; Hedges et al. 2008; Frost 2015). It originated in the early to mid-Paleogene in Andean South America (Pinto-Sánchez et al. 2012; Pyron 2014) and represents the bulk of anuran diversity in the tropical Andes, a global biodiversity hotspot. The wide distributional range of *Pristimantis* could be a consequence of having terrestrial eggs, an evolutionary innovation that could allow the colonization of new ecological niches unreachable for frogs that depend on water for their reproduction (Gomez-Mestre et al. 2012). The diversity of *Pristimantis* is strikingly higher than closely related clades (i.e. *Oreobates* with 23 species and *Lynchius* with 4 species; Frost 2015; Gonzalez-Voyer et al. 2011; Pyron and Wiens 2001).

*Pristimantis* is notorious for its taxonomic problems (e.g., Arroyo et al. 2005) perhaps as a consequence of its high diversity and morphologic plasticity (i.e. skin texture, color pattern, width of finger discs; Hedges et al. 2008; Guayasamin et al. 2015). As in most metazoans, its alpha-taxonomy is mainly based on morphological characters (e.g. Lynch and Duellman 1997). The advent of molecular systematics facilitated the discovery of cryptic diversity and the reinterpretation of morphological variation. Nevertheless, unexplored regions in the Andes still harbor many non-cryptic species that are demonstrably new even without genetic information.

A region where amphibian inventories have been almost completely lacking is Llanganates National Park in the central Andes of Ecuador. With an area of 2197 km^2^, Llanganates is a mosaic of páramos and montane forests dominated by a complex topography that result in a great diversity of habitats (Ministerio de Ambiente del Ecuador 2013). Efforts to survey its amphibian fauna have been sparse, partly as a consequence of the inaccessibility of most areas in the park. Field teams from Museo de Zoología at Catholic University of Ecuador visited the upper areas of the Park along the Salcedo-Tena road. The collections resulted in the discovery of two undescribed species of *Pristimantis* similar in morphology to *Pristimantis chloronotus*, *Pristimantis colonensis*, *Pristimantis eriphus* and *Pristimantis incanus*. In this publication we describe both species and evaluate their conservation status. We also present updated data for the species richness of Ecuadorian *Pristimantis* across biogeographic regions and analyze their possible environmental correlates.

## Materials and methods

### Morphology

The format for the descriptions follows Lynch and Duellman (1997). The terminology and definition of diagnostic characters follows Duellman and Lehr (2009). Specimens were preserved in 10% formalin and stored in 70% ethanol. Sex was determined by the presence of vocal slits and by direct gonadal inspection. Measurements were taken with digital calipers and rounded to the nearest 0.1 mm. We measured SVL (snout–vent length), TL (tibia length), FL (foot length, distance from proximal margin of inner metatarsal tubercle to tip of Toe IV), HL (head length, obliquely from angle of jaw to tip of snout), HW (head width, at level of angle of jaw), ED (eye diameter, distance between the anterior and posterior borders of the visible eye), IOD (interorbital distance, distance between the medial edge of the orbits), EW (upper eyelid width, length of the visible eye along the outer edge of eyelid), IND (internarial distance, distance between the inner edges of nares), EN (eye–nostril distance, distance between the anterior corner of orbit and the posterior margin of nares). Fingers and toes are numbered preaxially to postaxially from I to IV and I to V, respectively. Comparative lengths of Toes III and V were determined when both were adpressed against Toe IV; lengths of Fingers I and II were compared when adpressed against each other. Examined specimens belong to the herpetological collection at Museo de Zoología, Pontificia Universidad Católica del Ecuador, Quito (QCAZ), and are listed in Suppl. material 1.

### Regional species richness

The most recent analysis of regional diversity of *Pristimantis* in Ecuador was published by Lynch and Duellman (1997). Since then, the number of species has increased by ~50%. Herein we present an update on the patterns of regional diversity of *Pristimantis* in Ecuador.

Species richness values by biogeographic region are based on the AmphibiaWebEcuador database (http://zoologia.puce.edu.ec/Vertebrados/anfibios; Ron et al. 2016). To understand what variables could explain species richness across biogeographic regions, we correlated species number with area, mean annual precipitation, mean annual temperature, and median elevation. We chose these variables because previous analyses have shown that area and climate are significantly correlated with species richness in Ecuadorian Amphibians (Ron et al. 2011) and also worldwide (Pyron and Wiens 2013). Mean annual precipitation and mean annual temperature were obtained from random points over digital climate maps downloaded from WorldClim (http://www.worldclim.org). Median elevation was obtained from Sierra et al. (1999).

## Results

### Systematic accounts

#### Pristimantis yanezi sp. n.

Taxon classificationAnimaliaAnuraCraugastoridae

http://zoobank.org/C9B3FBF2-B1E3-4C44-B87E-CBF83349BDCA

##### Common name.

English: Yánez Rain Frog. Spanish: Cutín de Yánez.

##### Holotype

QCAZ 46259 (field no. SC-PUCE 29819; Figs 2A–B, 3A–B, 4A–B), adult male from Ecuador, Provincia Napo, Cantón Tena, on the road from Salcedo to Tena (1.0090°S, 78.1883°W), 2095 m, collected by Elicio E. Tapia and Fernando Núñez on 17 November 2009.

##### Paratopotypes

**(2 specimens).** QCAZ 46257 adult female, 46258 adult male, collected with the holotype.

##### Paratype

**(1 specimen).** QCAZ 45964 adult male from Ecuador, Provincia Pastaza, Cantón Santa Clara, Communal Reserve Ankaku (1.2792° S, 78.0779° W), 2280 m, collected by Elicio E. Tapia on 24 October 2009.

##### Diagnosis.

The new species is assigned to the genus *Pristimantis*. Although morphological synapomorphies are unknown for *Pristimantis*, the new species has the characteristic morphology of most *Pristimantis* including T-shaped terminal phalanges, toes without membranes, and Toe V longer than Toe III. *Pristimantis yanezi* is characterized by the following combination of characters: (1) Skin on dorsum smooth in the anterior half and shagreen or tuberculate in the posterior half, skin on venter areolate to weakly areolate; discoidal fold absent; dorsolateral folds absent; (2) tympanic membrane and tympanic annulus present, its upper and posterior margin covered by supratympanic fold; (3) snout short, rounded in dorsal and lateral view; (4) upper eyelid with one distinct conical tubercle surrounded by some low indistinct rounded tubercles; EW 92% of IOD; cranial crests absent; (5) dentigerous processes of vomers prominent, oblique, moderately separated, posteromedial to choanae; (6) vocals slits and nuptial pads absent; (7) Finger I shorter than Finger II; discs of digits expanded, truncate; (8) fingers without lateral fringes; (9) ulnar and carpal tubercles present, low and rounded; (10) heel bearing one low conical tubercle surrounded or not by few lower rounded tubercles; inner tarsal fold present, short; (11) inner metatarsal tubercle elliptical, prominent, 3X as large as outer metatarsal tubercle; outer metatarsal tubercle small, ovoid; low, numerous distinct supernumerary plantar tubercles; (12) toes without lateral fringes; basal toe webbing absent; Toe V slightly longer than Toe III (disc on Toe III reaches the middle of the penultimate subarticular tubercle on Toe IV, disc on Toe V does not reach the subarticular tubercle on Toe IV); toe discs about as large as those on fingers; (13) in life, dorsum yellowish brown to dark brown with scattered pale brown or orange blotches and black flecks, bearing a faint middorsal hourglass-shaped band; head bearing a dark brown interorbital bar and sides of head brown with darker vertical labial bars; flanks dark brown or olive brown with distinct dark brown to black flecks and diffuse dark brown diagonal stripes; groins cream or brownish cream; venter light cream to dirty cream with dark brown flecks and with or without dark brown mottling on the throat; iris reddish coppery; (14) SVL in adult female 36.9 mm (*n* = 1), in adult males 23.7–29.8 mm (*n* = 3).

##### Comparison with other species.

**Figure 1.**
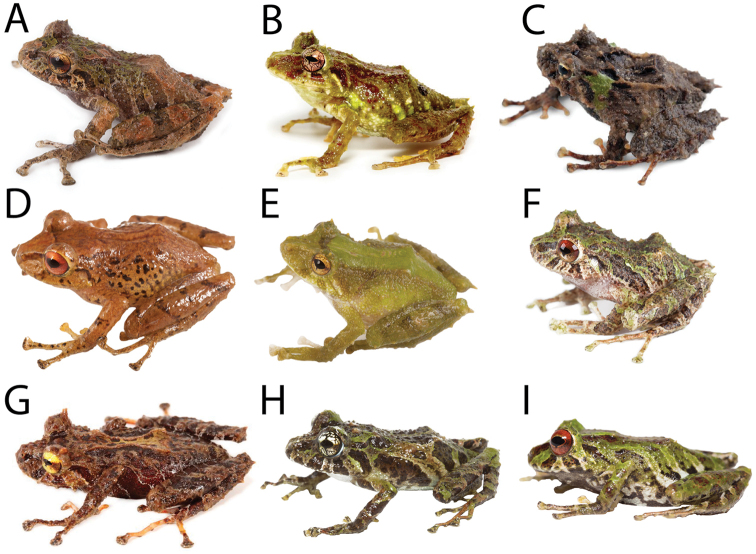
Coloration in life of new species and similar congeners. **A** *Pristimantis llanganati* sp. n., QCAZ 46227, adult male, SVL = 24.0 mm **B** *Pristimantis roni*, QCAZ 58928, adult male, SVL = 27.5 mm **C** *Pristimantis bellae*, QCAZ 46253, adult male, SVL = 22.0 mm **D** *Pristimantis yanezi* sp. n., QCAZ 45964, adult male, SVL = 27.8 mm **E** *Pristimantis inusitatus*, QCAZ 40107, SVL = no voucher available **F** *Pristimantis crucifer*, QCAZ 56765, adult female, SVL = 25.6 mm **G** *Pristimantis colonensis*, QCAZ 53318, adult female, SVL = 28.8 mm **H** *Pristimantis katoptroides*, QCAZ 58896, adult male, SVL = 18.9 mm **I** *Pristimantis eriphus*, QCAZ 58603, adult male, SVL = 23.2 mm. Pictures are not scaled.

In this section, coloration refers to live individuals unless otherwise noted. *Pristimantis yanezi* is similar to congeneric species characterized by a spiny appearance (i.e. presence of conical tubercles on dorsum, eyelids, heels and outer edge of tarsus). It differs from these species and from other *Pristimantis* by the combination of the following characters: iris reddish coopery, dorsum yellowish brown to brown bearing scattered pale brown to orange blotches; skin on flanks shagreen with small scattered tubercles and bearing distinctive brown to black flecks; upper eyelid, heel and outer edge of tarsus with small conical tubercles; venter and throat cream to dirty cream covered by brown flecks or brown mottling; groins cream or brownish cream; posterior surfaces of thighs and concealed surfaces of shanks brown or olive brown. Adult males of *Pristimantis yanezi* can be distinguished from *Pristimantis chloronotus* (Lynch 1969), *Pristimantis colonensis* (Mueses-Cisneros 2007), *Pristimantis crucifer* (Boulenger 1899), *Pristimantis eriphus* (Lynch and Duellman 1980), *Pristimantis galdi* (Jiménez de la Espada 1870), *Pristimantis inusitatus* (Lynch and Duellman 1980), *Pristimantis llanganati* sp. n., *Pristimantis mutabilis* Guayasamin, Krynak, Krynak, Culebras, and Hutter 2015, *Pristimantis rufoviridis* Valencia, Yánez-Muñoz, Betancourt-Yépez, Terán-Valdez, and Guayasamin 2011, *Pristimantis roni* Yánez-Muñoz, Bejarano-Muñoz, Brito M., and Batallas 2014, and *Pristimantis verecundus* (Lynch and Burrowes 1990) in lacking vocal slits. *Pristimantis bellae* Reyes-Puig and Yánez-Muñoz 2012, *Pristimantis colonensis*, *Pristimantis inusitatus*, *Pristimantis roni*, and *Pristimantis rufoviridis* also differ from *Pristimantis yanezi* by having a prominent conical tubercle on the eyelids and heels (conical tubercle is small in *Pristimantis yanezi*; Fig. 1). Furthermore, *Pristimantis bellae* has the groins, anterior and posterior surfaces of thighs, and concealed surfaces of shanks black with white spots or blotches (groins are cream or brownish cream and posterior surfaces of thighs and concealed surfaces of shanks are brown or olive brown with scattered faint cream flecks in *Pristimantis yanezi*). *Pristimantis colonensis* further differs from *Pristimantis yanezi* in having narrow white diagonal stripes on flanks (flanks with faint dark brown diagonal stripes in *Pristimantis yanezi*; Fig. 1). In dorsal view, *Pristimantis inusitatus* has the snout subacuminate with a pointed tip, while in *Pristimantis galdi* and *Pristimantis rufoviridis* the snout is acuminate (snout is rounded in *Pristimantis yanezi*). *Pristimantis roni* has lateral fringes on fingers and toes (absent in *Pristimantis yanezi*). *Pristimantis chloronotus*, *Pristimantis eriphus*, and *Pristimantis llanganati* sp. n. can be easily distinguished from *Pristimantis yanezi* by having flanks with clear and dark diagonal bars (flanks without bars in *Pristimantis yanezi*). Additionally, *Pristimantis chloronotus* has a pair of sinuous paravertebral folds (absent in *Pristimantis yanezi*). Furthermore, *Pristimantis eriphus* has the dorsum covered by many minute conical tubercles (dorsum smooth to shagreen in *Pristimantis yanezi*). *Pristimantis incanus* (Lynch and Duellman 1980) can be easily distinguished from *Pristimantis yanezi* by having groins yellow to light green or red (reddish brown in ethanol) with contrasting light or dark marks (groins are cream to tan cream without contrasting marks in *Pristimantis yanezi*). *Pristimantis crucifer* and *Pristimantis katoptroides* (Flores, 1988) differ from *Pristimantis yanezi* by having blue groins (cream or brownish cream in the new species). Both species also differ in iris coloration: red in *Pristimantis crucifer* and cream with black reticulations in *Pristimantis katoptroides* (iris reddish coopery in *Pristimantis yanezi*). Finally, *Pristimantis yanezi* differs from *Pristimantis mutabilis* and *Pristimantis verecundus* in lacking dorsolateral folds and red coloration in the groins.

##### Description of the holotype.

Adult male. Measurements (in mm): SVL 29.8; tibia length 17.0; foot length 14.8; head length 8.6; head width 11.5; eye diameter 3.7; tympanum diameter 1.1; interorbital distance 3.4; upper eyelid width 3.1; internarial distance 2.7; eye–nostril distance 3.3; tympanum–eye distance 1.5. Head wider than long, wide as body; head width 39% of SVL; head length 29% of SVL; snout rounded in dorsal view and in profile; eye–nostril distance 87% of eye diameter; nostrils narrow, higher than long, directed dorsolaterally; canthus rostralis distinct in lateral view, curved in dorsal view; loreal region concave; lips rounded; upper eyelid bearing one small but distinct conical tubercle surrounded by few indistinct smaller tubercles; upper eyelid width 92% of IOD; tympanic annulus distinct, with upper and posterior margins covered by supratympanic fold; tympanic membrane present, distinct; tympanum diameter 30% of eye diameter, tympanum–eye distance 134% of tympanum diameter; one enlarged conical postrictal tubercle surrounded by indistinct low tubercles. Choanae large, semicircular, not concealed by palatal shelf of maxilla; dentigerous processes of vomers prominent, oblique, moderately separated, positioned posteromedial to choanae; each vomer bearing several indistinct teeth; vocal slits absent; tongue two times wider than long, notched behind, free posteriorly along one third of its length.

**Figure 2.**
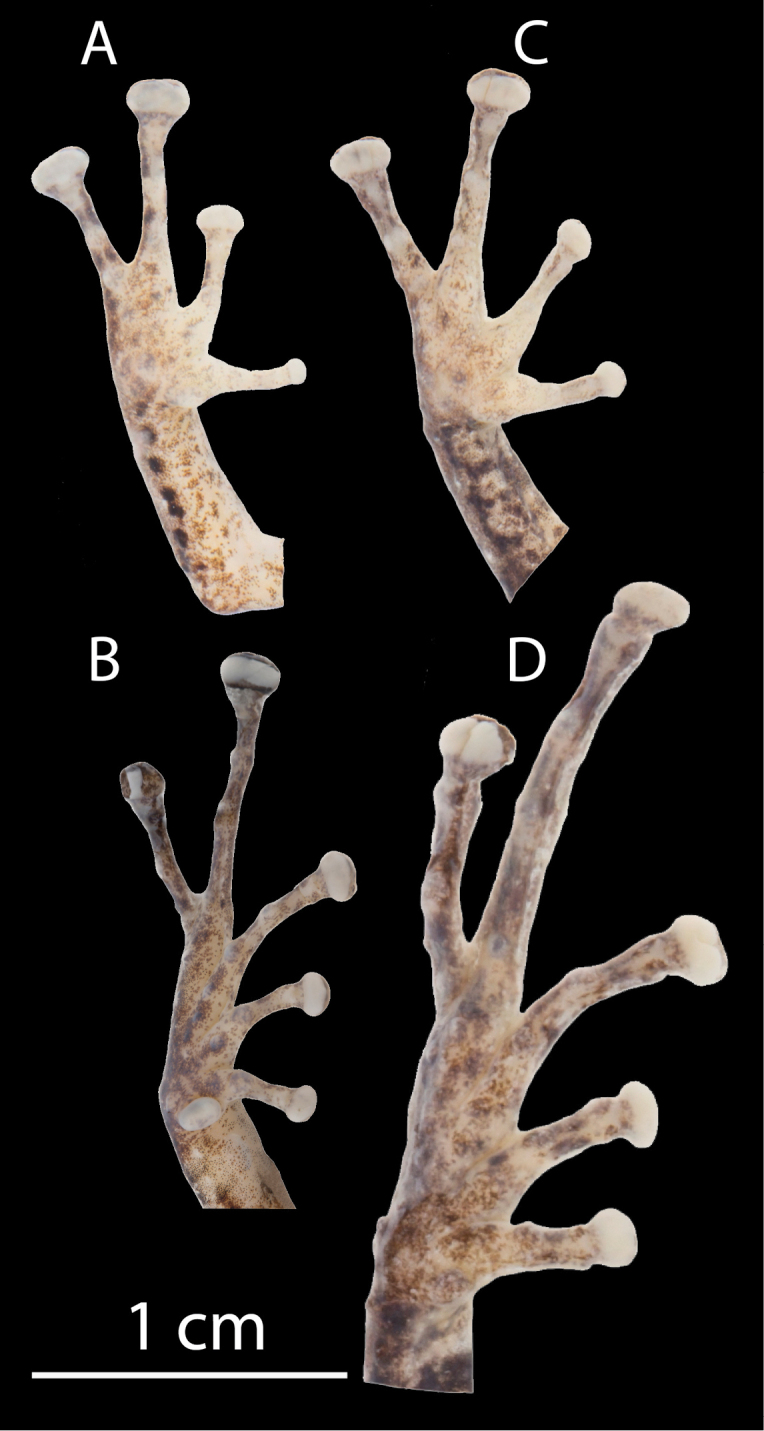
Palmar and plantar surfaces of the new species. Photos of hand (**A**) and foot (**B**) of *Pristimantis yanezi* sp. n., QCAZ 46259 (holotype), adult male, Hand Length (HL) = 11.4 mm, Foot Length (FL) = 14.8; hand (**C**) and foot (**D**) of *Pristimantis llanganati* sp. n., QCAZ 46140 (holotype), adult male, HL = 10.2 mm, FL = 22.8 mm.

Skin on dorsum smooth in the anterior half and shagreen in the posterior half; dorsolateral folds absent; skin on flanks with scattered tubercles; skin on throat, chest and belly weakly areolate, ventral surfaces of thighs areolate; discoidal fold absent; cloacal sheath short; skin in upper cloacal region shagreen, wrinkled ventrally, with several tubercles below the cloacal sheath. Ulnar tubercles present, indistinct; nuptial pads absent; palmar tubercles low, outer palmar tubercle bifid, approximately twice size of ovoid thenar tubercle; subarticular tubercles low, well defined, round in ventral and lateral view; supernumerary tubercles at base of fingers present, distinct; fingers lacking lateral fringes; Finger I shorter than Finger II; disc on Finger I rounded and on Finger II expanded, disc on Finger III and Finger IV broadly expanded and truncate; pads on fingers well defined, surrounded by circumferential grooves on all fingers (Fig. 2).

Hindlimbs slender, tibia length 57% of SVL; foot length 50% of SVL; upper surfaces of hindlimbs smooth; posterior surfaces of thighs smooth, ventral surfaces of thighs areolate; heel bearing one low conical tubercle surrounded by some low rounded tubercles; outer surface of tarsus bearing low but distinct sub-conical tubercles; short inner tarsal fold present; inner metatarsal tubercle prominent, elliptical, rounded, much bigger than oval, ill-defined outer metatarsal tubercle; plantar surface with some supernumerary tubercles; subarticular tubercles well defined, round in ventral and lateral view; toes lacking lateral fringes; webbing between toes absent; discs nearly as large as those on fingers, most prominent on Toe IV and V; discs on toes expanded, elliptical; all Toes having pads surrounded by circumferential grooves, less distinct on Toe I; relative lengths of toes: 1 < 2 < 3 < 5 < 4 (Fig. 2); Toe V longer than Toe III (disc on Toe III reaches the middle of the penultimate subarticular tubercle on Toe IV, disc on Toe V extends to proximal edge of distal subarticular tubercle on Toe IV).

**Figure 3.**
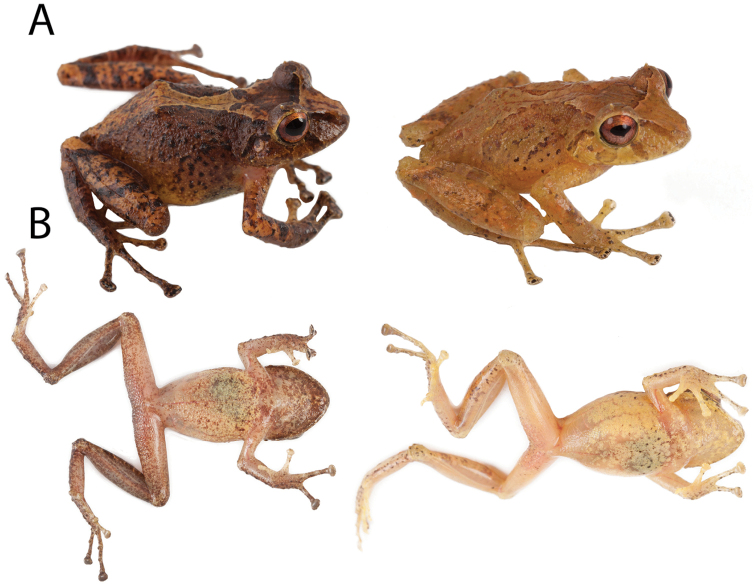
Coloration in life of *Pristimantis yanezi* sp. n. **A** dorsal view **B** ventral view. From left to right: QCAZ 46257, adult female, SVL = 36.9 mm; QCAZ 46259 (Holotype), adult male, SVL = 29.8 mm. Pictures are not scaled.

Color of holotype in life (based on digital photographs) (Fig. 3): dorsal surfaces of body, limbs, fingers and toes olive brown bearing a faint mid-dorsal hourglass-shaped band paler than the rest of dorsum, with brown flecks in the posterior half; top of the head, anterior to dark brown interorbital stripe, paler than the rest of head and dorsum; brown canthal stripe; two brown labial bars below orbit; flanks with faint brown diagonal stripes and scattered dark brown flecks, groins with a pale brown blotch; dorsal surfaces of forelimbs bearing dark brown flecks and diffuse brown bands; dorsal surfaces of thighs with faint brown bars and posterior surfaces of thighs olive brown; shanks, tarsus and feet bearing scattered brown flecks. Ventral areas of body, limbs, palms and soles yellowish cream with faint cream mottling on the throat and belly, scattered dark brown flecks on chest and belly, palms and soles. Iris reddish coppery.

**Figure 4.**
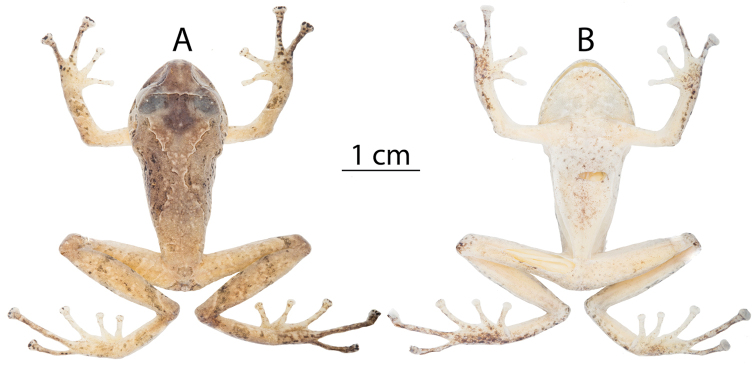
Preserved holotype of *Pristimantis yanezi* sp. n., QCAZ 46259, adult male, SVL = 29.8 mm. Dorsal (**A**), ventral (**B**) views.

Color of holotype in ethanol 70% (Fig. 4): dorsal surfaces of body, limbs, fingers and toes pale grayish brown, hourglass-shaped middorsal band is paler than the rest of dorsum, head is dusty brown darker than dorsum, the interorbital stripe is bright cream and the occipital region dark brown; sides of head dusty brown with dark brown canthal stripe and labial bars; flanks paler than dorsum with diffuse brown diagonal stripes and scattered brown flecks, groins with a pale cream blotch; dorsal surfaces of forelimbs bearing dark brown flecks, especially on fingers, and diffuse brown bands; dorsal surfaces of thighs with indistinct faint brown bars and posterior surfaces of thighs creamy brown with faint pale flecks; shanks, tarsus, and feet darker than thighs bearing scattered brown flecks and faint brown bands. Ventral areas of body, limbs, palms, and soles dirty cream with faint cream mottling on the throat and belly, scattered dark brown flecks on chest, belly, thighs, palms, and soles.

##### Variation.

**Figure 5.**
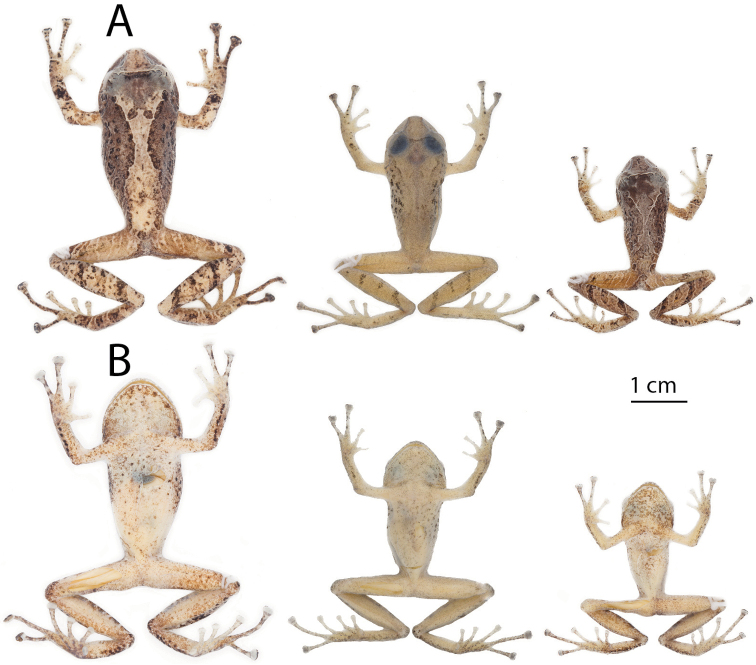
Preserved individuals of *Pristimantis yanezi* sp. n. showing dorsal and ventral variation. **A** dorsal view **B** ventral view. From left to right: QCAZ 46257, adult female, SVL = 36.9 mm; QCAZ 45964, adult male, SVL = 27.8 mm; QCAZ 46258, adult male, SVL = 23.7 mm. All the specimens are shown at the same scale.

In this section, coloration refers to preserved individuals. In the type series, adult males (23.7–29.8 mm) are smaller than the single known female (SVL = 36.9 mm). See Table 1 for measurements and proportions of the type specimens. Males lack vocals slits and nuptial pads. The middorsal hourglass-shaped band can be ill defined (QCAZ 46258) or absent (QCAZ 45964) (Fig. 5). Background coloration varies from brown or dark brown to olive yellow. Marks on dorsum and flanks are similar in all paratypes, except for the interorbital bar, that can be broad (QCAZ 46257) or narrow (QCAZ 45964) and brown (QCAZ 46257) or cream (QCAZ 46258). In life and preservative (Fig, 3; Fig. 5, respectively), the top of head anterior to the orbits can be darker than the rest of dorsum, except in QCAZ 46258 whose head and dorsum are uniform dark brown.

**Table 1.** Measurements (in mm) and proportions of type series of *Pristimantis yanezi* sp. n. Ranges followed by means and one standard deviation in parentheses. All specimens are adults.

Characters	Females (n = 1)	Males (n = 3)
SVL	36.9	23.7–29.8 (27.1 ± 3.1)
TL	18.5	13.5–17 (15.6 ± 1.8)
FL	17.3	12.5–14.8 (13.7 ± 1.2)
HL	10.4	7.6–8.6 (8.3 ± 0.5)
HW	14.2	9.4–11.5 (10.55 ± 1.1)
ED	4.6	3.2–3.7 (3.5 ± 0.3)
TY	1.4	0.9–1.3 (1.1 ± 0.2)
IOD	3.3	2.8–3.4 (3.0 ± 0.3)
EW	3.8	2.6–3.1 (2.9 ± 0.3)
IND	3	2.2–2.3 (2.4 ± 0.3)
E–N	3.9	2.2–3.3 (2.9 ± 0.6)
TL/SVL	0.5	0.6
FL/SVL	0.5	0.9
HL/SVL	0.3	0.3
HW/SVL	0.4	0.4
HW/HL	1.4	1.2–1.3 (1.3 ± 0.1)
E–N/ED	0.9	0.7–0.9 (0.8 ± 0.1)
EW/IOD	1.2	0.9–1.0 (1.0 ± 0.1)
TY/ED	0.3	0.3

Coloration in life (based on digital photographs of adult female QCAZ 46257 and of male QCAZ 45964) (Fig. 1; Fig. 3): dorsum dark olive yellow to brown with scattered dark brown flecks, two dark brown (QCAZ 46257) or pale brown (QCAZ 45964) scapular spots, dorsum bearing an hourglass-shaped mark in the mid-dorsum (less defined in QCAZ 45964); head with dark brown interorbital bar; sides of head brown with dark brown canthal stripe and brown labial bars, flanks dark brown with black flecks, groins with creamy yellow blotches; dorsal surface of forelimbs dirty light brown with dark brown marks and dark brown flecks on fingers; dorsal surfaces of thighs brown with faint (QCAZ 45964) or conspicuous (QCAZ 46257) brown diagonal bars; shanks light brown with dark brown diagonal stripes, tarsus and feet dirty brown with dark brown bars (QCAZ 46257) or dark brown blotches (QCAZ 45964). Throat brownish cream, chest and belly cream with scattered dark brown flecks; ventral surfaces of thighs, shanks, and tarsus brownish cream; palmar and plantar surfaces brown. Iris reddish copper. Sexual dimorphism in morphology could not be evaluated due to the limited sample size (n = 4; one female and three males).

##### Distribution, natural history, and conservation status.

*Pristimantis yanezi* is known from two localities (elevation range is 2095–2280 m) from Provincia del Tungurahua and Provincia del Pastaza, Parque Nacional Llanganates. Airline distance between localities is 32 km. Ecosystem type is Evergreen Montane Forest of the Eastern Andean Cordillera (as defined by Ministerio de Ambiente del Ecuador 2013) or Eastern Montane Forest (as defined by Ron et al. 2016).

The holotype and the paratopotypes were collected at night, on vegetation on recently logged forest. The paratype was collected at night, on a branch (1 cm diameter) 2 m above the ground. A deforestation map by Ministerio de Ambiente (2013) shows continuous forest at the known localities. Because we lack population data and most of the Llanganates region lacks amphibian inventories, we assign *Pristimantis yanezi* to the Data Deficient Red List category (based on IUCN 2001 guidelines).

##### Etymology.

The specific name *yanezi* is a noun in the genitive case and is a patronym for Mario Yánez who provided useful insights for the description of the new species. Moreover, during his career, Mario Yánez has contributed significantly to the study of Ecuadorian amphibians, especially those of the genus *Pristimantis*. He is director of Museo Ecuatoriano de Ciencias Naturales (MECN).

##### Remarks.

Most species groups within *Pristimantis* have been defined exclusively on morphological grounds (e.g., Lynch and Duellman 1997). With few exceptions, those groups resulted artificial (i.e., non-monophyletic) once phylogenies based on genetic characters were used to evaluate them (e.g., Pinto-Sanchez et al. 2012). Because morphological characters in *Pristimantis* are unreliable to assess phylogenetic affinities, we refrain from assigning *Pristimantis yanezi* to a species group.

#### Pristimantis llanganati sp. n.

Taxon classificationAnimaliaAnuraCraugastoridae

http://zoobank.org/4B160419-87CB-48AE-85BF-B66C263A9B18

##### Common name.

English: Llanganates Rain Frog. Spanish: Cutín de los Llanganates.

##### Holotype

**Figure 6.**
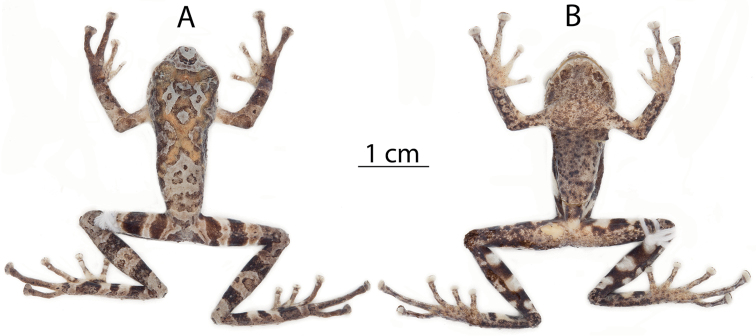
Preserved holotype of *Pristimantis llanganati* sp. n., QCAZ 46140, adult male, SVL = 27.1 mm. Dorsal (**A**), ventral (**B**).

QCAZ 46140 (field no. SC-PUCE 29720; Figs 2C–D, 6A–B), adult male from Ecuador, Provincia Napo, Cantón Tena, “La Cueva” at the confluence of the Mulatos and Langoa rivers (0.9663° S, 78.2224° W), 2483 m above the sea, collected by Elicio E. Tapia and Fernando Núñez on 15 November 2009.

##### Paratopotypes

**(3 specimens).** QCAZ 46221 adult female, 46141 and 46142 juveniles, collected with the holotype.

##### Paratypes

**(2 specimens).** Ecuador, Provincia Napo, QCAZ 46227 adult male, Salcedo-Tena road (0.9847°S, 78.1928°W), 2253 m, collected by Elicio E. Tapia and Fernando Núñez on 16 November 2009; QCAZ 46217, juvenile, Salcedo-Tena road (0.9670°S, 78.2484°W), 2883 m, collected by Elicio E. Tapia and Fernando Núñez on 14 November 2009

##### Diagnosis.

The new species is assigned to the genus *Pristimantis*. Although morphological synapomorphies are unknown for *Pristimantis*, the new species has the characteristic morphology of most *Pristimantis* including T-shaped terminal phalanges, toes without membranes, and Toe V longer than Toe III. *Pristimantis llanganati* is characterized by the following combination of characters: (1) Skin on dorsum covered by minute conical tubercles, skin on venter areolate with scattered warts; discoidal fold absent; dorsolateral folds absent; (2) tympanic membrane and tympanic annulus present, covered by supratympanic fold on its upper and posterior margins; (3) snout short, rounded in dorsal and lateral view; (4) upper eyelid with a low conical tubercle and some low indistinct tubercles posteriorly; EW 82% of IOD; cranial crests absent; (5) dentigerous processes of vomers varying from low and indistinct to high and evident, oblique, moderately separated, posteromedial to choanae; (6) vocals slits present, nuptial pads absent; (7) Finger I shorter than Finger II; discs of digits broadly expanded, truncate; (8) fingers bearing narrow lateral fringes; (9) ulnar and tarsal tubercles present, conical and low; (10) heel with two or three low conical tubercles; inner tarsal fold present, long and ill defined; (11) inner metatarsal tubercle elliptical, low, 2X as large as outer metatarsal tubercle; outer metatarsal tubercle small, ovoid; supernumerary plantar tubercles low and indistinct; (12) toes bearing narrow lateral fringes; toe webbing absent; Toe V longer than Toe III (disc on Toe III reaches the proximal edge to the proximal subarticular tubercle on Toe IV, disc on Toe V extends to the proximal edge of distal subarticular tubercle on Toe IV); toe discs about as large as those on fingers; (13) in life, dorsum olive green with X-shaped or rhomboidal dark brown mark on scapular region, and scattered brown flecks or blotches; flanks, dorsal and posterior surfaces of thighs and ventral surfaces of shanks dirty white or white with dark brown diagonal stripes; groins white or tan with distinct black or dark brown diagonal stripes; venter dirty cream with brown mottling in the throat and chest and brown flecks in belly and ventral surfaces of thighs. Iris coppery with a reddish horizontal stripe; (14) SVL in one adult female 29.8 mm, in adult males 24.0–27.0 mm (*n* = 2).

##### Comparisons with other species.

(Fig. 1). In this section, coloration refers to live individuals unless otherwise noted. *Pristimantis llanganati* can be easily distinguished from other congeners from the Andes of Ecuador and Colombia, except *Pristimantis chloronotus*, *Pristimantis colonensis*, and *Pristimantis eriphus*, by the presence of the following traits: dorsum green, greenish brown or mossy; spiny appearance bearing distinct conical tubercles on eyelids, heels and outer edge of tarsus; groins white or tan with distinct black or dark brown diagonal stripes; posterior surfaces of thighs and concealed surfaces of shanks with oblique white and brown or black bars. *Pristimantis llanganati* is most similar to *Pristimantis eriphus*. Both species have greenish or mossy coloration and tuberculate skin. However, *Pristimantis eriphus* can be readily distinguished from *Pristimantis llanganati* in having a brown vertebral band or dark brown chevrons on the scapular regions (brown or reddish brown X-shaped or rhomboidal mark in *Pristimantis llanganati*) and greenish coopery or red iris without reticulations (copper iris with dark brown reticulations in *Pristimantis llanganati*). *Pristimantis chloronotus* and *Pristimantis colonensis* can be easily distinguished from *Pristimantis llanganati* in having sinuous paravertebral folds (absent in *Pristimantis llanganati*; Fig. 1). In addition, *Pristimantis chloronotus* upper eyelids are covered with small conical tubercles (one distinct conical tubercle surrounded by several lower conical tubercles in *Pristimantis llanganati*). *Pristimantis colonensis* also differs from *Pristimantis llanganati* in having the posterior surfaces of thighs dark brown with white or cream oblique lines (white with brown bars in *Pristimantis llanganati*), and iris yellowish gold (coppery in *Pristimantis llanganati*). *Pristimantis incanus* is also similar to *Pristimantis llanganati* in coloration and disposition of tubercles on eyelids, heels, and tarsus. However, males of *Pristimantis incanus* haven vocal slits (absent in *Pristimantis llanganati*). Moreover, *Pristimantis incanus* have glossy white points on groins, posterior surfaces of thighs and concealed surfaces of shanks (those areas are white and black or have dark brown stripes in *Pristimantis llanganati*).

##### Description of the holotype.

Adult male. Measurements (in mm): SVL 27.0; tibia length 18.8; foot length 22.8; head length 7.9; head width 9.8; eye diameter 3.2; tympanum diameter 1.3; interorbital distance 3.4; upper eyelid width 2.7; internarial distance 2.3; eye–nostril distance 2.8; tympanum–eye distance 1.3. Slender body; head as wide as body, wider than long; head width 36% of SVL; head length 29% of SVL; snout short, rounded in dorsal and lateral view; eye–nostril distance 89% of eye diameter; nostrils narrow, higher than long, directed dorsolaterally; canthus rostralis curved in dorsal view, slightly curved in profile; loreal region concave; lips rounded; upper eyelid bearing one small conical tubercle on its center and some low rounded tubercles posteriorly; upper eyelid width 82% of IOD; tympanic annulus barely visible, with upper and posterior margins covered by supratympanic fold; tympanic membrane present, distinct; tympanum diameter 39% of eye diameter, tympanum–eye distance 102% of tympanum diameter; few indistinct postrictal tubercles present. Choanae small, semicircular, not concealed by palatal shelf of maxilla; dentigerous processes of vomers low, indistinct, oblique, moderately separated, posteromedial to choanae; right vomer bearing two teeth and left vomer one tooth; vocal slits present; tongue slightly wider than long, notched behind, free posteriorly along one third of its length.

Skin on dorsum covered by minute conical tubercles, head bearing two large tubercles, one between the orbits and the other half way between the interorbital line and the tip of the snout; dorsolateral folds absent; skin on flanks with minute conical tubercles; skin on throat and chest weakly areolate, belly areolate with scattered enlarged warts, and ventral surfaces of thighs weakly areolate; discoidal fold absent; cloacal sheath short; skin in cloacal region tuberculate with two enlarged tubercles, on each side, below the cloacal sheath. Ulnar tubercles present, subconical and low; elbow bearing one low subconical tubercle; nuptial pads absent; palmar tubercles low, weakly defined, outer palmar tubercle bifid, approximately twice the size of ovoid thenar tubercle; subarticular tubercles low, well defined, round in ventral and lateral view; supernumerary tubercles at base of fingers present, indistinct; fingers bearing narrow lateral fringes; Finger I shorter than Finger II; disc on Finger I rounded and on Finger II expanded, disc on Finger III and IV broadly expanded and truncate; pads on fingers well defined by circumferential grooves on all fingers (Fig. 2).

Hindlimbs slender, tibia length 70% of SVL; foot length 84% of SVL; upper surfaces of hindlimbs with minute conical and subconical tubercles; posterior surfaces of thighs smooth, ventral surfaces weakly areolate; knee bearing low conical tubercles; heel bearing low conical tubercles; inner tarsal fold present, long and weakly defined; inner metatarsal tubercle low, elliptical, rounded, two times the size of smaller, oval, rounded outer metatarsal tubercle; plantar surface weakly tuberculate; subarticular tubercles well defined, round in ventral and lateral view; toes bearing narrow lateral fringes; basal webbing between toes absent; discs nearly as large as those on fingers, most prominent on Toe IV and V; discs on toes expanded, rounded; all toes having ventral pads well defined by circumferential grooves; relative lengths of toes: 1 < 2 < 3 < 5 < 4 (Fig. 2); Toe V longer than Toe III (disc on Toe III reaches the proximal edge of the proximal subarticular tubercle on Toe IV, disc on Toe V reaches the proximal edge of distal subarticular tubercle on Toe IV).

Color of holotype in life is unknown. Color of holotype in ethanol 70% (Fig. 6): dorsal background color is greenish gray with a U-shaped brown mark on the top of head, a broad reddish-brown interorbital bar, an X-shaped reddish brown mark on the mid-dorsum, and scattered brown blotches; sides of head with a broad dark brown canthal stripe, two brown vertical labial bars, brown supratympanic fold; flanks white with dark brown diagonal stripes; groins white with dark brown diagonal stripes; forelimbs with brown transversal bars, hindlimbs with dark brown (nearly black) diagonal transversal bars; posterior surfaces of thighs and concealed surfaces of shanks whitish cream with broad dark brown bars. Venter creamy brown with brown mottling on throat, chest, and forelimbs, dark brown flecks on belly and ventral surfaces of thighs; palms and soles brownish cream.

##### Variation.

**Figure 7.**
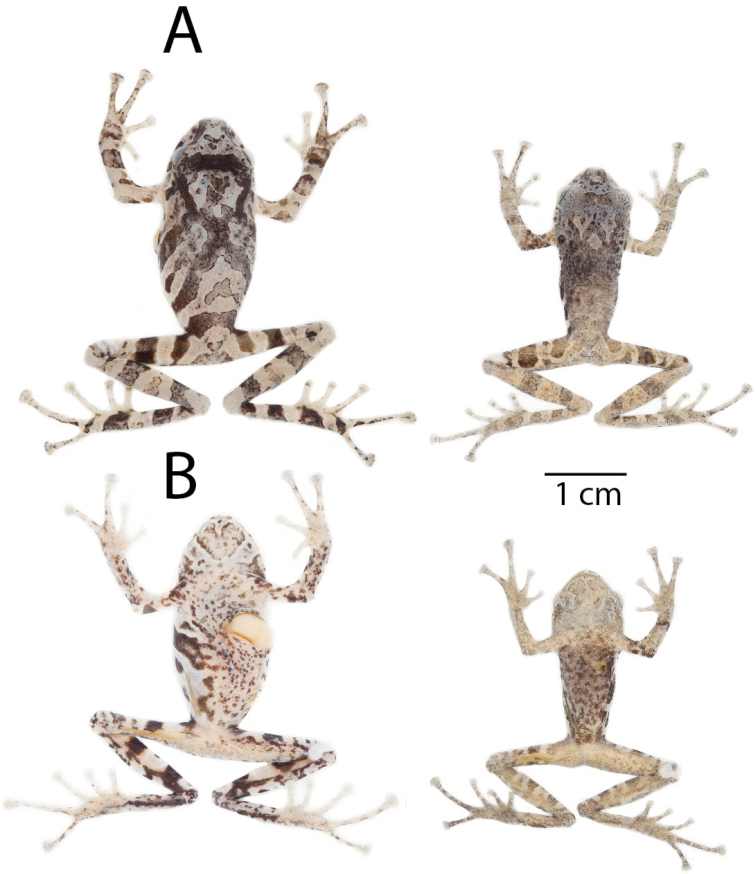
Preserved individuals of *Pristimantis llanganati* sp. n. showing dorsal and ventral variation. **A** dorsal view **B** ventral view. From left to right: QCAZ 46221, adult female, SVL = 29.8 mm; QCAZ 46227, adult male, SVL = 24.0 mm. Specimens are shown at the same scale.

In this section, coloration refers to preserved individuals. In the type series, adult males (24.0–27.0 mm) (n = 2) are smaller than the single known female (SVL = 29.8 mm) (n = 1). See Table 2 for measurements and proportions of the type specimens and see Figure 7 for photographs of preserved individuals. The single adult male paratype (QCAZ 46227) differs from the holotype in having less conspicuous dorsal marks. The only known female (QCAZ 46221) has the dorsum grayish cream. Three juvenile specimens (QCAZ 46217, SVL 19.3 mm; QCAZ 46141, SVL 12.5 mm and QCAZ 46142, SVL 10.7 mm) are identical in coloration to the holotype but lack well defined dorsal marks. The juvenile QCAZ 46142 has a dark brown venter.

**Table 2.** Measurements (in mm) and proportions of the type series of *Pristimantis llanganati* sp. n. All specimens are adults, range and average (in parenthesis) show for two males.

Characters	Females (n = 1)	Males (n = 2)
SVL	29.8	24.0–27.0 (25.0)
TL	16.3	13.2–18.8 (16.0)
FL	15.7	18.2–22 8 (20.5)
HL	8.2	7.2–7.9 (7.5)
HW	10.3	9.0–9.8 (9.4)
ED	3.5	2.9–3.2 (3.04)
TY	1.2	0.9–1.1 (1.0)
IOD	3.4	2.7–3.4(3.0)
EW	3.2	2.36–2.7 (2.5)
IND	2.3	2.2–2.3 (2.2)
E–N	3.1	2.4–2.8 (2.6)
TL/SVL	0.6	0.6–0.7 (0.6)
FL/SVL	0.5	0.8
HL/SVL	0.3	0.3
HW/SVL	0.4	0.4
HW/HL	1.3	1.3
E–N/ED	0.9	0.9
EW/IOD	0.9	0.8
TY/ED	0.3	0.3

**Figure 8.**
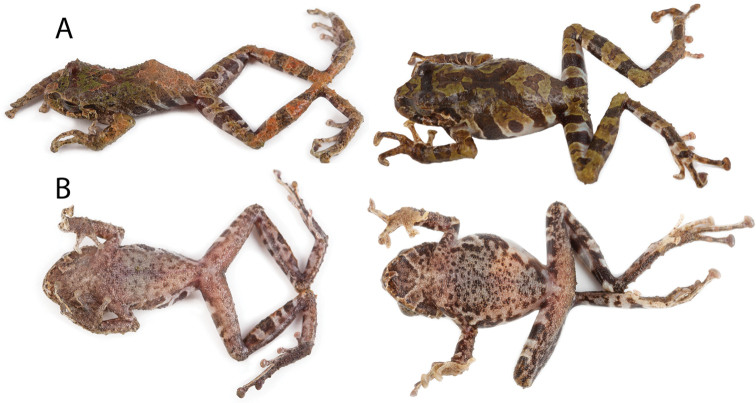
Coloration in life of *Pristimantis llanganati* sp. n. **A** dorsal view **B** ventral view. From left to right: QCAZ 46227, adult male, SVL = 24.0 mm; QCAZ 46221, adult female, SVL = 29.8 mm. Pictures are not scaled.

Coloration in life (Fig. 8): Based on digital photographs of adult male (QCAZ 46227) and of adult female (QCAZ 46221). Dorsum olive green with an X-shaped mark in the mid-dorsum (QCAZ 46221) or with a light brown blotch on the scapular region with a rhomboidal dark brown mark and dark brown flecks (QCAZ 46227) limbs bearing transversal broad dark brown bars, posterior and anterior surfaces of thighs and concealed surfaces of shanks white with dark brown bars; top of head light brown to brown with a broad dark brown interorbital bar; sides of head greenish brown with a dark brown canthal stripe, two broad dark brown labial bars and dark brown supratympanic fold; flanks, including the groins white to whitish cream with broad dark brown diagonal stripes; anterior and posterior surfaces of thighs, concealed surfaces of shanks and dorsal surfaces of limbs are white with dark brown transversal bars. Ventrally, throat and chest are dirty cream with brown mottling, belly and ventral surfaces of thighs are dirty cream with brown flecks; ventral surfaces of tarsus, forelimbs, palms, and soles brown with minute cream flecks. Iris cooper with dark brown reticulations and a reddish midhorizontal band. Sexual dimorphism in morphology could not be evaluated due to the small sample of adult individuals (n = 3; one female and two males).

##### Distribution, natural history, and conservation status.

*Pristimantis llanganati* is known from three localities (elevation range is 2253–2883 m) from Provincia del Napo, Parque Nacional Llanganates, along the Salcedo-Tena road. Maximum airline distance between localities is 6.5 km. Ecosystem type is Evergreen Montane Forest of the Eastern Andean Cordillera (as defined by Ministerio de Ambiente del Ecuador 2013) or Eastern Montane Forest (as defined by Ron et al. 2016).

All specimens were collected at night on vegetation. Four of them were in a flooded area in forest border. Two were in primary forest. A deforestation map by Ministerio de Ambiente (2013) shows continuous forest at the known localities. Because we lack population data and most of the Llanganates region is unexplored, we assign *Pristimantis llanganati* to the Data Deficient Red List category (based on IUCN 2001 guidelines).

**Figure 9.**
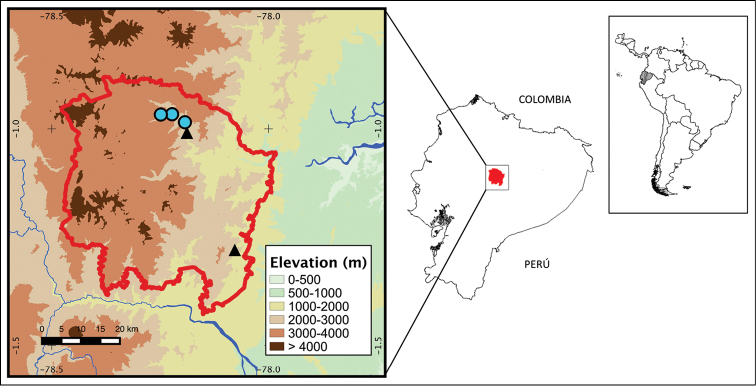
Map showing known localities for *Pristimantis llanganati* sp. n. and *Pristimantis yanezi* sp. n. Circles are for *Pristimantis llanganati* sp. n. and triangles for *Pristimantis yanezi* sp. n. Localities are based on type specimens deposited at the QCAZ collection (see Suppl. material 1 for a list). The limit of Llanganates National Park is shown in red.

##### Etymology.

The species name *llanganati* is a noun that refers to the kichwa word “Llanganati” that means “beautiful hill”. This word also gives name to Llanganates National Park, the area where the species was discovered. Many areas in the park are difficult to access and are biologically unexplored. This inaccessibility has protected large areas of its páramos and montane forests, a valuable asset for the conservation and Andean biodiversity.

##### Remarks.

As in *Pristimantis yanezi*, *Pristimantis llanganati* is not assigned to a species group until genetic data allows determining its phylogenetic position.

### Regional species richness

**Figure 10.**
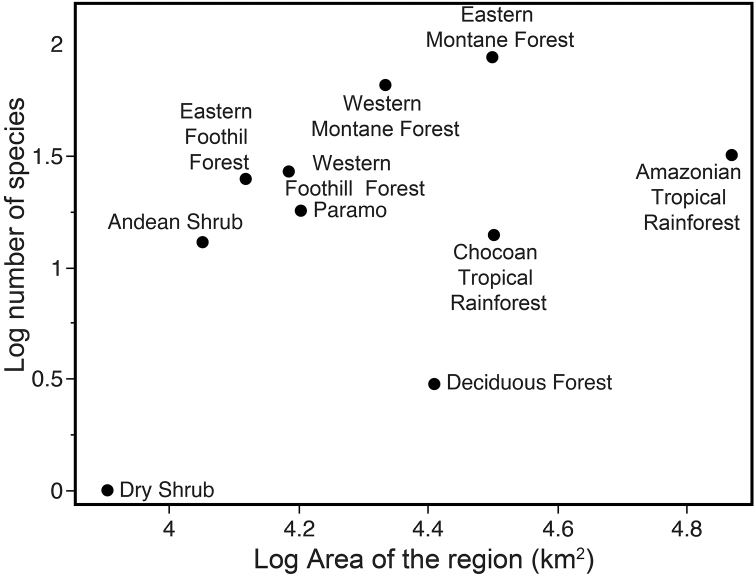
Relationship between *Pristimantis* species richness and geographic area for Ecuadorian Biogeographic Regions (as shown in Fig. 9). The regression line is shown in red. Dry habitats in the lowlands (Dry Shrub and Deciduous Forest) show lower species richness than what is predicted from their geographic extent.

Across biogeographic regions (Fig. 10), *Pristimantis* diversity peaks in the Eastern Montane Forest (88 species) and Western Montane Forest (66 species; Fig. 10). The regions with the lowest richness are the dry habitats in the lowlands: Costal Shrub (1 species) and Tropical Deciduous Forest (3 species). The Amazonian Tropical Rainforest has 32 species, a modest richness considering its large size (29.8% of Ecuador’s area).

**Figure 11.**
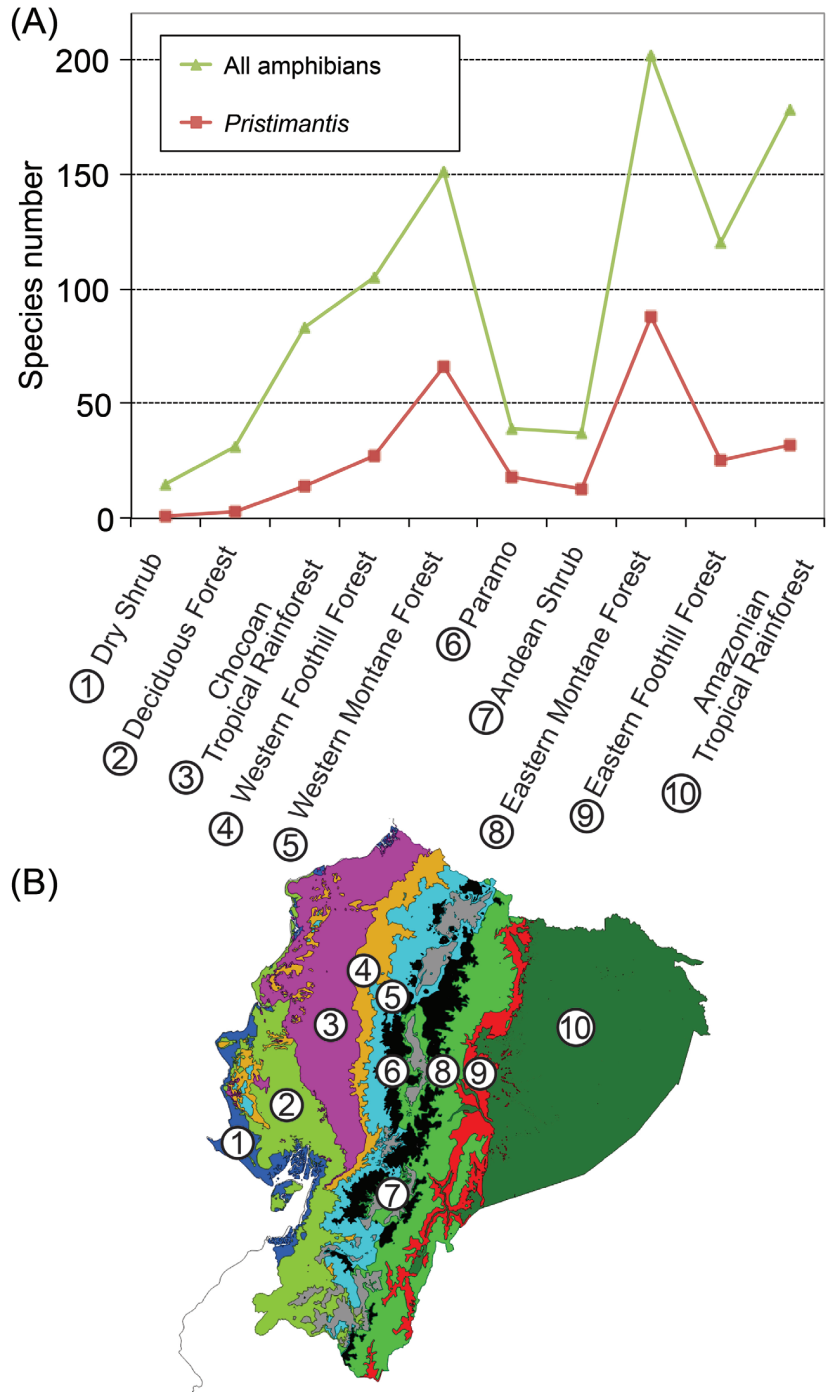
**A** Species richness of all amphibians (triangles) and *Pristimantis* (squares) across 10 biogeographic regions in Ecuador **B** Map of the biogeographic regions (based on Ron et al. 2016). Numbers in the map correspond to those of the regions in (**A**).

Relative to all Ecuadorian amphibians, *Pristimantis* represents a higher proportion of the amphibian fauna in Páramo (46.1%) and montane forests (43.7% in Western Montane, 43.6 in Eastern Montane; Fig. 10). They are a minor component of communities in Dry Shrub (6.7%), Deciduous Forest (9.7%), and Chocoan Tropical Rainforest (16.9%). Ninety-eight species of *Pristimantis* are endemic to Ecuador (54% of the total). Surprisingly, the correlation between species richness and area of the region was not significant (*F* = 2.21, *df* = 9, *P* = 0.176) (Fig. 11). Precipitation, temperature, and elevation were not correlated with species richness either (*P* values 0.136, 0.225, and 0.167, respectively).

## Discussion

### Regional species richness

During the last decades, the number of described species of Ecuadorian amphibians has been steadily increasing reaching 566 species by 2016 (Ron et al. 2016). Of them, the most speciose genus is *Pristimantis* with 184 species. The two new species described here occur in the Andean Montane Forest, the Ecuadorian region where *Pristimantis* reach their highest diversity (Ron et al. 2016). Our results indicate that *Pristimantis* regional richness is highest on montane forests and lowest in the dry lowlands. A similar pattern has been reported in Peru where the majority of *Pristimantis* occur in humid forests in the Andes and few species occur in the dry lowland habitats west of the Andes (Duellman and Lehr 2009). The most striking disparity in species richness between Ecuador and Peru occurs at the highest elevations in the Andes. Despite being much less extensive, the Ecuadorian Páramo has 18 species of *Pristimantis* in contrast to the five species reported for the Peruvian Puna by Duellman and Lehr (2009). Diversity across biogeographic regions in Ecuador is consistent with the high diversity of Craugastoridae and *Pristimantis* reported in Andean forests in general (Hedges et al. 2008; Duellman and Lehr 2009).

The proportion of Pristimantis species endemic to Ecuador (98 spp., 54% of all Pristimantis) compares with 41% country endemism across all amphibians (Ron et al. 2016), 2% among birds (Navarrete 2010), 10% among mammals (Tirira 2007), and 25% among reptiles (Torres-Carvajal et al. 2015). A high political endemism likely results from the relatively narrow distributions that characterize most species of *Terrarana*.

Lack of correlation between species richness and area of the region seems to be a consequence of the extremely low species richness of the Dry Shrub and Deciduous Forest and the extremely high richness in montane forests, relative to their size. Precipitation, temperature, and elevation were not correlated either. These results differ from local and global analyses indicating that environmental variables are strongly correlated with amphibian species richness (Ron et al. 2011, Pyron and Wiens 2013). Lack of correlation could be a real pattern or an artifact of the large number of species that remain undescribed in the Andes.

In summary, Ecuadorian *Pristimantis* reach their highest diversity in Montane Forests of the eastern versant of the Andes. Relative to other amphibian groups, they are particularly diverse in the Páramo region. Its species richness across regions cannot be explained by regional area, elevation, temperature, or precipitation.

## Supplementary Material

XML Treatment for Pristimantis yanezi

XML Treatment for Pristimantis llanganati

## Supplementary materials

Supplementary material 1 List of specimens examined and associated collection data Data type: Occurrence Explanation note: All samples belong to genus *Pristimantis*. We present museum and field number, locality, and coordinates, for each individual. QCAZ = Museo de Zoología de la Pontificia Universidad Católica del Ecuador . Abbreviations in the locality column are: BI = Bosque integral ; BS = Biological station ; CR = Communal reserve ; NP = National Park . This dataset is made available under the Open Database License (http://opendatacommons.org/licenses/odbl/1.0/). The Open Database License (ODbL) is a license agreement intended to allow users to freely share, modify, and use this Dataset while maintaining this same freedom for others, provided that the original source and author(s) are credited.María J. Navarrete, Pablo J. Venegas, and Santiago R. Ron
